# Predictive factors for the relapse medication overuse headache. A prospective study

**DOI:** 10.1186/1129-2377-14-S1-P173

**Published:** 2013-02-21

**Authors:** A Sergeev, M Mesherina, G Tabeeva

**Affiliations:** 1 Sechenov First Moscow State Medical University, Russian Federation

## Introduction

The prevalence of medication overuse headache (MOH) in population is 1-2 percent, in headache centers is 60-70 percent [1,2]. The MOH relapse during the 1st year after therapy occurs in 30-45 percent [3].

## Objectives

Prospective study of predictive factors of MOH relapse after drug withdrawal and prophylactic therapy.

## Methods

Trial was performed in 45 patients with MOH in age from 18 to 65 years (mean age 55,±9,9 years). All patients underwent abuse drug withdrawal and the course of preventive therapy. Prospective analysis of clinical-psychological characteristics at 2, 6 and 12 months was performed.

## Results

Predictors of treatment effectiveness and return to episodic headache after 1 year are initially low intensity of headache, low dose of an analgesic per month (<30 dose per month), the absence of drug dependence and of high level of depression. Predictors of MOH relapse after 1 year are impossibility of simultaneous withdrawal of the abuse drug, prolonged course of disease, early age of debut, combination of comorbid disorders (depression, anxiety, personal and mood disorders), high level of analgesic dependency, analgesics containing barbiturates.

## Conclusion

The hypothesis of heterogeneity of the MOH was confirmed. The 1st type is MOH without comorbid psychiatric disorders with high level of effectiveness of preventive therapy. The 2nd type is MOH with comorbid psychiatric disorders (depression, anxiety, personal and mood disorders), drug dependence and low efficiency of preventive therapy. The choice of strategy of preventive therapy should be implemented depending on the type of MOH and identified predictors of relapse.

## Conflict of interest

The authors declare that they have no conflict of interest related to the publication of this manuscript.
